# Association of the angiotensin I converting enzyme (*ACE*) gene polymorphisms with recurrent aphthous stomatitis in the Czech population: case–control study

**DOI:** 10.1186/s12903-022-02115-3

**Published:** 2022-03-19

**Authors:** Julie Bartakova, Tereza Deissova, Simona Slezakova, Jirina Bartova, Jitka Petanova, Pavel Kuklinek, Antonin Fassmann, Petra Borilova Linhartova, Ladislav Dušek, Lydie Izakovicova Holla

**Affiliations:** 1 Clinic of Stomatology, Institution Shared with St. Anne’s University Hospital, Faculty of Medicine, Masaryk University, Pekarska 664/53, 60200 Brno, Czech Republic; 2 Department of Pathophysiology, Faculty of Medicine, Masaryk University, Kamenice 753/5, 62500 Brno, Czech Republic; 3 Department of Biochemistry, Faculty of Science, Masaryk University, Kamenice 753/5, 62500 Brno, Czech Republic; 4 Czech National Centre for Evidence-Based Healthcare and Knowledge Translation (Cochrane Czech Republic, Czech EBHC: JBI Centre of Excellence, Masaryk University GRADE Centre), Institute of Biostatistics and Analyses, Faculty of Medicine, Masaryk University, Kamenice 753/5, 62500 Brno, Czech Republic; 5 Department of Stomatology, General University Hospital in Prague and First Faculty of Medicine, Charles University, Karlovo nam. 554/32, 12808 Prague, Czech Republic; 6 Institute of Immunology and Microbiology, General University Hospital in Prague and First Faculty of Medicine, Charles University, Karlovo nam. 554/32, Prague, Czech Republic; 7 Department of Clinical Immunology and Allergology, Institution Shared with St. Anne’s Faculty Hospital and Faculty of Medicine, Masaryk University, Pekarska 664/53, 60200 Brno, Czech Republic; 8 RECETOX, Faculty of Science, Masaryk University, Kotlarska 2, 61137 Brno, Czech Republic; 9 Institute of Biostatistics and Analyses, Faculty of Medicine, Masaryk University, Kamenice 753/5, 62500 Brno, Czech Republic

**Keywords:** Recurrent aphthous stomatitis, Angiotensin I converting enzyme, Polymorphism, Haplotype, Sex difference

## Abstract

**Background:**

Recurrent aphthous stomatitis (RAS) is multifactorial disease with unclear etiopathogenesis. The aim of this study was to determine distribution of the angiotensin I converting enzyme (*ACE)* gene polymorphisms and their influence on RAS susceptibility in Czech population.

**Methods:**

The study included 230 subjects (143 healthy controls and 87 patients with RAS) with anamnestic, clinical and laboratory data. Five *ACE* gene polymorphisms (rs4291/rs4305/rs4311/rs4331/rs1799752 = *ACE* I/D) were determined by TaqMan technique.

**Results:**

The allele and genotype distributions of the studied *ACE* I/D polymorphisms were not significantly different between subjects with/without RAS (*P*_corr_ > 0.05). However, carriers of II genotype were less frequent in the RAS group (OR = 0.48, 95% CI = 0.21–1.12, *P* = 0.059). Stratified analysis by sex demonstrated lower frequency of II genotype in women (OR = 0.33, 95% CI = 0.09–1.17, *P* < 0.035, *P*_corr_ > 0.05, respectively) than in men with RAS (*P* > 0.05). Moreover, the frequency of AGTGD haplotype was significantly increased in RAS patients (OR = 13.74, 95% CI = 1.70–110.79, *P* = 0.0012, *P*_corr_ < 0.05). In subanalysis, TGD haplotype was significantly more frequent in RAS patients (*P* < 0.00001) and CGI haplotype was less frequent in RAS patients (*P* < 0.01), especially in women (*P* = 0.016, *P*_corr_ > 0.05).

**Conclusions:**

Our study indicates that while the AGTGD and TGD haplotypes are associated with increased risk of RAS development, CGI haplotype might be one of protective factors against RAS susceptibility in Czech population.

## Background

Recurrent aphthous stomatitis (RAS) is a chronic multifactorial disease characterized by the presence of recurrent painful erosions or ulcers on the oral mucosa. Although exact etiopathogenesis of RAS is uncertain, several factors such as local trauma, stress, nutrition, hormonal changes, hypersensitivity and microbial factors have been implicated in this disease [1]. Besides them, genetic background can also play a role [2, 3]; it has been found that > 40% RAS patients have a familial history [4].

One of the candidate genes for RAS encodes angiotensin I converting enzyme (ACE). This zinc metallopeptidase is a regulatory component of the renin–angiotensin (RA) system by hydrolyzing angiotensin I (Ang I) to angiotensin II (Ang II) and inactivating the bradykinin [5]. Ang II not only increases blood pressure but is also a potent proinflammatory modulator which through the production of reactive oxygen species can induce tissue damage. Besides systemic RA, the local renin–angiotensin system contributes to the inflammatory process via stimulation of the production of cytokines [6].

The *ACE* gene is mapped on chromosome 17q23.3 and contains a number of variable polymorphic regions with possible functional implications. More than half of the inter-individual variability in ACE levels is a consequence of polymorphism (rs1799752) that consists of the presence (insertion, I) or absence (deletion, D) of a 287-bp Alu repeat sequence in intron 16 of this gene. The I allele is associated with lower enzyme activity compared with the D allele [7]. The location of this polymorphism in a non-coding region of the gene, however, makes it unlikely to be a functional variant. Despite considerable efforts, the precise location of the functional polymorphisms is still unknown [8]. Previously, the *ACE* polymorphisms (including the *ACE* I/D polymorphism) and ACE plasma levels were analyzed in Caucasian British families. Due to strong linkage disequilibrium (LD) operating over this small chromosomal region where the *ACE* gene is located, the analysis of polymorphisms revealed a limited number of haplotypes [9]. Alterations in the *ACE* gene have been associated with different multifactorial diseases with inflammatory background and the presence of oral ulcers as one of the symptoms such as Behçet's disease (BD) [10, 11]. In addition, case–control study in a Turkish population suggested that the *ACE* I/D polymorphism in intron 16 might affect RAS development [12].

The aim of our study was to determine the distribution of *ACE* gene polymorphisms and their influence on RAS susceptibility in the Czech population.

## Methods

### Study design, clinical examination and sample collection

This case–control genetic association study was conducted in the period of from 2014 to 2018. Individuals were recruited from pools of Clinic of Stomatology, Institution Shared with St. Anne's Faculty Hospital and Faculty of Medicine, Masaryk University, Brno, Czech Republic and from Institute of Immunology and Microbiology, General University Hospital and First Faculty of Medicine, Charles University, Prague, Czech Republic.

The diagnosis of RAS was based on the generally accepted criteria [13]. The inclusion criteria were the presence of aphthous lesions examined and diagnosed by an oral medicine specialist and recurring episodes of aphthous ulcers according to patient’s history. RAS was further divided into three types according to Karakus et al. [12]: (1) minor (less than 1 cm in diameter, healing within 10–14 days, (2) major (larger than 1 cm and deeper than the minor form, healing within 10–30 days, (3) herpetiform aphthae (grouped aphthae, 1–2 mm in size). The exclusion criteria included the presence of any local oral disease or systemic disorder with oral manifestations including BD, celiac disease, and the use of immunomodulatory drugs or systemic steroids. To exclude systemic disorders, the routine biochemical (e.g. glucose, liver function tests), haematological (e.g. blood count with differential, red blood cell folate assay, ferritine levels, vitamin B12), serological tests (e.g. anti-herpes simplex virus antibodies) and immunological tests (e.g. ASCA IgA and IgG and ANCA) were performed. The control group was recruited from systematically healthy individuals without history of RAS and the above-mentioned exclusion criteria.

The study protocol was approved by the Committees for Ethics of Masaryk University, Faculty of Medicine (39/2015), General University Hospital and First Faculty of Medicine, Charles University, Prague (53/14) and St. Anne's Faculty Hospital Brno (8G/2015). Written informed consent was obtained from the study participants in line with the Declaration of Helsinki prior to their inclusion in the study.

### Isolation of genomic DNA and genetic analysis

Genomic DNA was purified from peripheral blood leukocytes by the standard method using the phenol–chloroform extraction and proteinase K digestion of cells.

Five *ACE* polymorphisms (rs4291, rs4305, rs4311, rs4331 and rs1799752 (I/D) polymorphism) were selected based on the study by Staalsø et al. [14]. These authors used the pairwise tagging algorithm in the Haploview 4.2 software [14] taking into account possible functional relevance of these polymorphisms [15–21] and minor allele frequency (MAF) in the European population (MAF higher than 10%).

Genotyping of the *ACE* I/D polymorphism (rs1799752) was based on polymerase chain reaction (PCR) using TaqMan^®^ assays with ABsolute QPCR Mix, ROX Thermo Fisher Scientific, Waltham, MA, USA) and primers designed by Koch et al. [22]. Allele genotyping from fluorescence measurements was then obtained using the ABI PRISM 7000 Sequence Detection System. SDS version 1.2.3 software was used to analyze real-time and end-point fluorescence data as described previously [23]. Genotyping of *ACE* A/T rs4291, *ACE* A/G rs4305, *ACE* C/T rs4311 and *ACE* A/G rs4331 were performed by quantitative PCR using 5′ nuclease TaqMan^®^ assays (C__11942507_10, C___1247703_20, C___1247707_1_, C__11942537_20). The reaction mixture and conditions were designed according to the manufacturer’s instructions (Thermo Fisher Scientific, Waltham, MA, USA) and fluorescence was measured using Roche LightCycler^®^ 96 System. The LightCycler^®^ 96 Application Software Version 1.1 was used to analyze real-time and endpoint fluorescence data. Genotyping was verified by rerunning ≥ 5% of the samples, which were 100% concordant.

### Statistical analysis

Power analysis was performed with respect to the case–control design of the study, taking the incidence rate of markers and the estimate of OR as end-point statistical measures. Absolute and relative frequencies for categorical variables and mean and standard deviation (SD) for quantitative variables were calculated. The allele frequencies were counted from the observed numbers of the genotypes by Fisher exact test. The chi-square test was used for analysis of Hardy–Weinberg equilibrium (HWE) and for comparison of the differences in the genotypes. Odds ratio (OR), confidence intervals (CI) and *P* values were calculated. All statistical analyses were performed using the program package Statistica v. 13 (StatSoft Inc., Tulsa, Okla., USA). The haplotype frequencies were calculated by SNP analyzer (http://snp.istech.info/istech/board/login_form.jsp). The problem of multiple hypothesis testing was corrected by Bonferroni method. The critical value (alpha) for an individual test was obtained by dividing the familywise error rate (0.05) by the number of tests. Correction due to multiple parameter testing was applied using standard computation based on the number of the dimensions involved. In the case of haplotype testing, we used an algorithm imbedded directly into the standardized SW toolkit (http://snp.istech.info/istech/board/login_form.jsp).

## Results

Two hundred and thirty Czech subjects were enrolled in this study: 143 healthy controls (65 males and 78 females; mean age ± SD: 47.6 ± 12.4 years) and 87 patients with RAS (34 males and 53 females; mean age ± SD: 39.0 ± 15.4 years). Their demographic data are shown in Table 1. The male/female distribution was not significantly different between both groups (*P* > 0.05), however, the healthy controls were statistically significantly older than the RAS patients (*P* < 0.01). Most of the patients with RAS (96.6%) suffered from minor apthae, only three patients had the major form. Almost 85% of the recruited RAS patients had at least four recurrences of oral erosions/ulcers per year (Table 1).

**Table 1:** Clinical characteristics of the examined subjects

Clinical parameters	Controls N = 143	RAS patients N = 87
Mean age (years ± SD)	47.6 ± 12.4	39.0 ± 15.4
Sex [N of men/women (%)]	65/78 (45.5/54.5)	34/53 (39.1/60.9)
Type of apthae		
Minor [N (%)]	NA	84 (96.6)
Major [N (%)]	NA	3 (3.4)
Herpetiform [N (%)]	NA	0 (0.0)
Mean number of lesions in each episode*		
< 3 lesions [N (%)]	NA	30 (35.3)
≥ 3 lesions [N (%)]	NA	55 (64.7)
Duration of lesions to healing*		
Less than 1 week [N (%)]	NA	43 (50.6)
Less than 2 weeks [N (%)]	NA	24 (28.2)
Two or more weeks [N (%)]	NA	18 (21.2)
Number of oral ulcer recurrences*		
Less than 3 times per year [N (%)]	NA	8/5 (9.4/5.9)
At least one per 3 months [N (%)]	NA	18 (21.2)
At least one per month [N (%)]	NA	15 (17.6)
At least 2 times per month [N (%)]	NA	12 (14.1)
Permanently [N (%)]	NA	27 (31.8)

*N* number of subjects, *RAS* recurrent aphthous stomatitis, *SD* standard deviation, *NA* non applicable

*In 2 patients this information was not available

The power of the study was set up with respect to Fisher exact test as the principal method comparing relative frequencies between groups and finally, with focus on quantitative estimate of OR. Given the recruited sample size, the test allowed to detect OR in the range of 0.5–2.3 as statistically significant at standard level of alpha = 0.05 and beta 0.80. The allele and genotype distributions of the *ACE* polymorphisms rs4291, rs4305, rs4311, rs4331 and rs1799752 (I/D) are presented in Table 2. The frequencies were in compliance with those expected by the HWE in the group of controls as well as in subgroups of healthy men and women (*P* > 0.05).

**Table 2:** *ACE* polymorphisms allele and genotype frequencies in patients with RAS and controls

*ACE*	Genotypes	Controls	RAS	*P* value	OR (CI 95%)	Controls	RAS	*P* value	OR (CI 95%)	Controls	RAS	*P* value	OR (CI 95%)
	Alleles	N = 143 (%)	N = 87 (%)			Men	Men			Women	Women		
						N = 65 (%)	N = 34 (%)			N = 78 (%)	N = 53 (%)		
rs4291	AA	59 (41.3)	33 (37.9)		1.00	30 (46.2)	18 (52.9)		1.00	29 (37.2)	15 (28.3)		1.00
	AT	70 (48.9)	40 (46.0)	0.365	0.98 (0.55–1.74)	31 (47.7)	12 (35.3)	0.392	0.65 (0.27–1.57)	39 (50.0)	28 (52.8)	0.462	1.39 (0.63–3.06)
	TT	14 (9.8)	14 (16.1)		1.79 (0.76–4.20)	4 (6.1)	4 (11.8)		1.67 (0.37–7.50)	10 (12.8)	10 (18.9)		1.93 (0.66–5.67)
	A	188 (65.7)	106 (60.9)	0.173	1.00	91 (70.0)	48 (70.6)	0.534	1.00	97 (62.2)	58 (54.7)	0.141	1.00
	T	98 (34.3)	68 (39.1)		1.23 (0.83–1.82)	39 (30.0)	20 (29.4)		0.97 (0.51–1.85)	59 (37.8)	48 (45.3)		1.36 (0.82–2.25)
rs4305	AA	25 (17.5)	17 (19.5)		1.33 (0.60–2.95)	11 (16.9)	6 (17.6)		1.13 (0.34–3.81)	14 (17.9)	11 (20.8)		1.89 (0.64–5.56)
	AG	73 (51.0)	47 (54.0)	0.712	1.26 (0.68–2.35)	33 (50.8)	15 (44.1)	0.803	0.73 (0.39–1.85)	40 (51.3)	32 (60.4)	0.312	1.92 (0.80–4.59)
	GG	45 (31.5)	23 (26.5)		1.00	21 (32.3)	13 (38.3)		1.00	24 (30.8)	10 (18.8)		1.00
	A	123 (43.0)	81 (46.6)	0.259	1.15 (0.79–1.69)	55 (42.3)	27 (39.7)	0.421	0.90 (0.49–1.63)	68 (43.6)	54 (50.9)	0.034*	1.66 (1.00–2.78)
	G	163 (57.0)	93 (53.4)		1.00	75 (57.7)	41 (60.3)		1.00	88 (56.4)	42 (39.6)		1.00
rs4311	CC	41 (28.7)	21 (24.1)		1.00	20 (30.8)	10 (29.4)		1.00	21 (26.9)	11 (20.8)		1.00
	CT	77 (53.8)	45 (51.8)	0.437	0.88 (0.46–1.67)	36 (55.4)	17 (50.0)	0.683	0.94 (0.36–2.45)	41 (52.6)	28 (52.8)	0.615	1.30 (0.54–3.12)
	TT	25 (17.5)	21 (24.1)		1.64 (0.75–3.59)	9 (13.8)	7 (20.6)		1.56 (0.45–5.41)	16 (20.5)	14 (26.4)		1.67 (0.60–4.65)
	C	159 (55.6)	87 (50.0)	0.142	1.00	76 (58.5)	37 (54.4)	0.346	1.00	83 (53.2)	50 (47.2)	0.202	1.00
	T	127 (44.4)	87 (50.0)		1.25 (0.86–1.83)	54 (41.5)	31 (45.6)		1.18 (0.65–2.13)	73 (46.8)	56 (52.8)		1.27 (0.78–2.09)
rs4331	AA	38 (26.6)	23 (26.4)		1.00	17 (26.2)	6 (17.6)		1.00	21 (26.9)	17 (32.1)		1.00
	AG	74 (51.7)	48 (55.2)	0.816	1.07 (0.57–2.02)	32 (49.2)	17 (50.0)	0.552	1.51 (0.50–4.53)	42 (53.9)	31 (58.5)	0.302	1.10 (0.50–2.42)
	GG	31 (21.7)	16 (18.4)		0.85 (0.39–1.89)	16 (24.6)	11 (32.4)		1.95 (0.58–6.51)	15 (19.2)	5 (9.4)		0.41 (0.12–1.36)
	A	150 (52.4)	94 (54.0)	0.408	1.00	66 (50.8)	29 (42.6)	0.175	1.00	84 (53.8)	65 (61.3)	0.142	1.00
	G	136 (47.6)	80 (46.0)		0.94 (0.64–1.37)	64 (49.2)	39 (57.4)		1.39 (0.77–2.50)	72 (46.2)	41 (38.7)		0.74 (0.45–1.22)
rs1799752 (I/D)	II	31 (21.7)	11 (12.6)		0.48 (0.21–1.12)	15 (23.1)	7 (20.6)		0.66 (0.21–2.11)	16 (20.5)	4 (7.5)		0.33 (0.09–1.17)
	ID	74 (51.7)	48 (55.2)	0.209	1.14 (0.62–2.09)	33 (50.8)	15 (44.1)	0.637	0.64 (0.25–1.68)	41 (52.6)	33 (62.3)	0.127	1.06 (0.48–2.34)
	DD	38 (26.6)	28 (32.2)		1.00	17 (26.1)	12 (35.3)		1.00	21 (26.9)	16 (30.2)		1.00
	I allele	136 (47.6)	70 (40.2)	0.075	0.74 (0.51–1.09)	63 (48.5)	29 (42.6)	0.265	0.79 (0.44–1.43)	73 (46.8)	41 (38.7)	0.120	0.72 (0.43–1.18)
	D allele	150 (52.4)	104 (59.8)		1.00	67 (51.5)	39 (57.4)		1.00	83 (53.2)	65 (61.3)		1.00

*N* number of subjects, *ACE* gene for angiotensin I converting enzyme, *D* deletion, *I* insertion, *RAS* recurrent aphthous stomatitis

*P_corr_ > 0.05 after correction for multiple comparisons

**Fig. 1:**
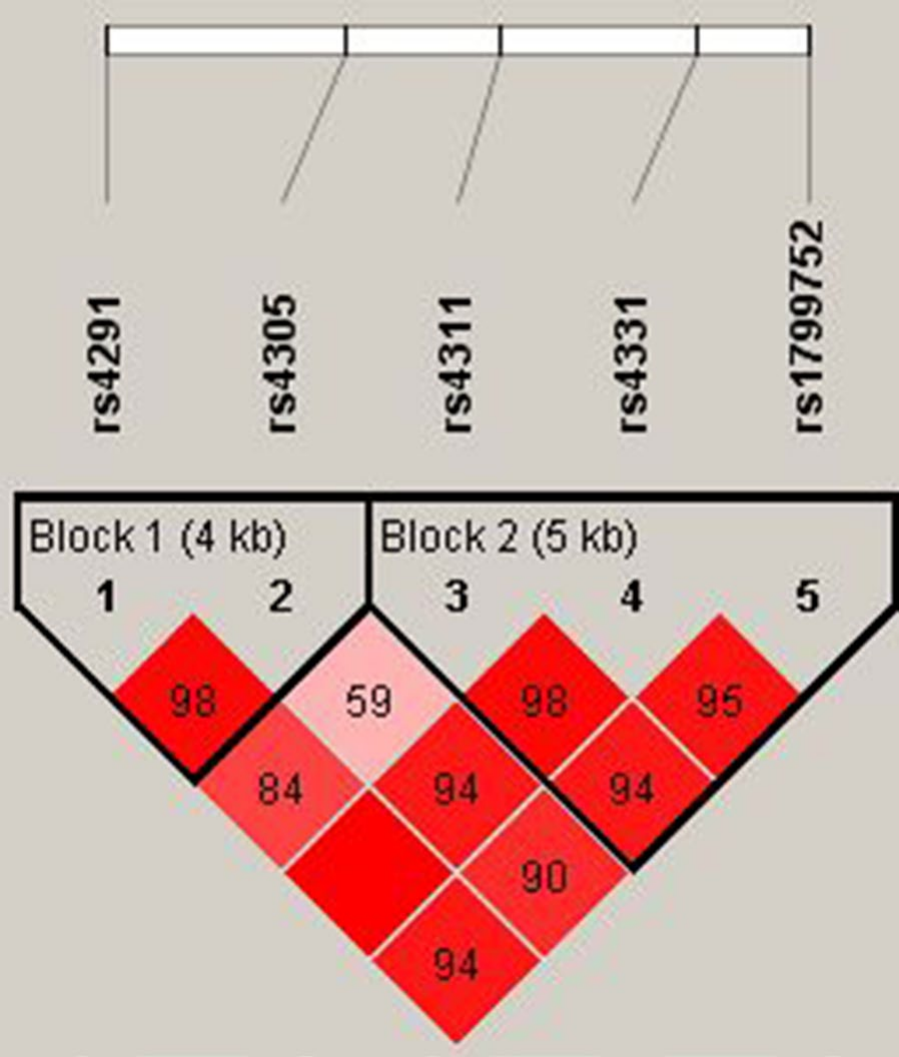
Linkage disequilibrium (LD) maps for 5 polymorphisms in the angiotensin I converting enzyme (*ACE*) gene. Strong LD is illustrated with red colour. LD blocks are marked by a black triangle (identified using the Solid spine of the LD method)

The pairwise LD was calculated, *ACE* polymorphisms rs4291 and rs4305 were in one LD block, while rs4311, rs4331 and rs1799752 (I/D) were in the other LD block (Fig. 1). Although the allele and genotype frequencies of the *ACE* polymorphisms rs4291, rs4305, rs4311, rs4331 and rs1799752 (I/D) between the groups of patients with RAS and healthy controls did not differ significantly, carriers of the II genotype of the *ACE* I/D polymorphism had a lower risk of RAS development than carriers of other genotypes (OR = 0.48, 95% CI = 0.21–1.12, *P* = 0.059).

Moreover, the frequency of haplotype AGTGD (rs4291/rs4305/rs4311/rs4331/rs1799752) was significantly increased in RAS patients in comparison to healthy controls (5.6% vs. 0.0%, OR = 13.74, 95% CI = 1.70–110.79, *P* = 0.0012, *P*_corr_ < 0.05). In subanalysis, the frequency of the haplotype TGD (rs4311/rs4331/rs1799752) was significantly higher in patients with RAS (10.5% vs. 0.4%, OR = 24.94, 95%CI = 3.25–191.40, *P* < 0.00001, *P*_corr_ < 0.001) and the haplotype CGI (rs4311/rs4331/rs1799752) was less frequent in patients with RAS (31.5% vs. 46.5%, OR = 0.58, 95%CI = 0.39–0.85, *P* < 0.01, *P*_corr_ < 0.05) (Table 3).

**Table 3:** *ACE* haplotypes frequencies in patients with RAS and controls

*ACE*	Haplotypes	Controls	RAS	*P* value	OR (CI 95%)	Controls	RAS	*P* value	OR (CI 95%)	Controls	RAS	*P* value	OR (CI 95%)
		N = 143 (%)	N = 87 (%)			Men	Men			Women	Women		
						N = 65 (%)	N = 34 (%)			N = 78 (%)	N = 53 (%)		
rs4311/rs4331/rs1799752 (I/D)	CGI	46.5	31.5*	0.005	0.575 (0.389–0.851)	48.5	36.6	0.168	0.658 (0.362–1.197)	44.9	28.5*	0.016	0.531 (0.316–0.895)
	TAD	43.0	36.5	0.479	0.871 (0.593–1.278)	41.5	27.0	0.139	0.629 (0.338–1.171)	44.2	42.6	0.866	1.043 (0.635–1.714)
	CAD	8.4	11.7	0.617	1.182 (0.616–2.269)	9.2	13.6	0.810	1.128 (0.423–3.013)	7.7	10.9	0.618	1.250 (0.519–3.008)
	TAI	1.1	0.0	NA	0 (0.000–0.000)	0.0	0.0	NA	0 (0.000–0.000)	1.9	0.0	NA	0 (0.000–0.000)
	CGD	0.7	1.0	0.150	3.341 (0.606–18.437)	0.8	2.1	0.093	5.954 (0.607–58.370)	0.7	0.0	0.784	1.476 (0.091–23.864)
	TGD	0.3	10.5*	< 0.00001	24.938 (3.249–191.404)	0.0	14.6*	NA	0 (0.000–0.000)	0.6	7.8*	0.012	9.300 (1.103–78.408)
	CAI	0.0	5.8	NA	0 (0.000–0.000)	0.0	2.0	NA	0 (0.000–0.000)	0.0	7.8	NA	0 (0.000–0.000)
	TGI	0.0	3.0	NA	0 (0.000–0.000)	0.0	4.0	NA	0 (0.000–0.000)	0.0	2.4	NA	0 (0.000–0.000)

*N* number of subjects, *ACE* gene for angiotensin I converting enzyme, *D* deletion, *I* insertion, *RAS* recurrent aphthous stomatitis

*P < 0.05, NA—non applicable (small—zero numbers)

A sex-stratified analysis demonstrated that the frequency of II genotype was lower in comparison with other genotypes in women (OR = 0.33, 95%CI = 0.09–1.17, *P* < 0.035, *P*_corr_ > 0.05, respectively). However, no significant differences among *ACE* alleles or genotypes in men with/without RAS were found (*P* > 0.05), (Table 2). In case of haplotypes, the haplotype CGI (rs4311/rs4331/rs1799752) was less frequent in women with RAS (28.5% vs. 44.9%, OR = 0.53, 95% CI: 0.32–0.90, *P* = 0.016, *P*_corr_ > 0.05), while the frequency of the haplotype TGD (rs4311/rs4331/rs1799752) was higher in women with RAS (7.8% vs. 0.6%, OR = 9.30, 95% CI: 1.10–78.41, *P* = 0.012, *P*_corr_ > 0.05; Table 3) than in healthy controls.

## Discussion

RAS is a very common disease; its etiopathogenesis involves complex interactions of genetic and environmental factors [2, 24]. The genetic control of immunity, cytokine production and inflammatory response led us to investigate the role of the *ACE* gene polymorphisms. I/D polymorphism in intron 16 belongs to the most investigated *ACE* gene variants. This polymorphism was primarily associated with cardiovascular diseases in several populations including Czech subjects [25, 26]. In addition, we also suggested the important role of the I/D *ACE* variant in atopic diseases [27], dental caries in permanent dentition [23] and marginally in chronic periodontitis [28]. However, we did not find any significant association between this polymorphism and pulmonary disease severity, fibrosis, and progression [29] or caries in primary dentition [23]. The variability in the *ACE* gene has previously been identified as a susceptibility factor for ulcers as one of the symptoms of BD in a Turkish population but with conflicting results [10, 11, 30, 31]. Although the *ACE* I/D polymorphism did not seem to play a role in etiopathogenesis of BD in the two smaller studies (N = 90 BD and N = 30 healthy controls, N = 73 BD and N = 90 controls) by Ozturk et al. [31] and Dursun et al. [30], Turgut and colleagues [10] found a statistically significant association of the *ACE* I/D polymorphism with BD in 35 patients and 150 healthy individuals. Similarly, a significant difference in frequencies of the *ACE* I/D alleles and genotype distribution between controls and patients with BD was found in the largest study of 566 subjects (266 patients and 300 healthy individuals) by Yigit and co-workers [11]. In addition, the *ACE* I/D polymorphism was significantly associated with RAS in Turkey where the authors found that the DD genotype and D allele were more common in RAS patients than in control subjects [12]. It is in agreement with our results of protectivity of II genotype of this polymorphism in the Czech population. Moreover, the frequency of the haplotype CGI (rs4311/rs4331/rs1799752) was less frequent in patients with RAS in our population. This haplotype contains C allele rs4311 and I allele rs1799752, which both were previously associated with lower ACE serum levels [14, 32, 33].

ACE has a significant role in inflammatory processes and is widely distributed in many tissues; some studies have reported that this enzyme can be expressed in the T-lymphocytes and ACE levels in these cells were significantly higher in the subjects who were homozygote for the deletion than in the others [32, 33]. Cellular immunity involves an important part of pathogenesis in RAS/BD; the damage in the oral mucosal epithelia in these diseases may result from immunological processes with a T-cell origin [34]. In addition, it has been demonstrated that *ACE* DD cells have higher levels of Ang II and are more prone to cell death than II cells. Ang II stimulation can lead to regulation of leukocyte extravasation, activation, chemotaxis, and proliferation of mononuclear cells and upregulation of proinflammatory mediators including cytokines and adhesion molecules [35]. As the levels of ACE are higher in DD carriers in comparison to II homozygotes, we speculate that the DD genotype has higher proinflammatory potential and may be associated with an increased risk of RAS than less active *ACE* II gene variant which, according to our results, can confer the protection against this disease. However, the direct mechanism responsible for the effect of different *ACE* alleles and/or genotypes on the development of RAS remains unclear.

Further, in this study, we investigated sex differences in the presence of *ACE* I/D allele and/or genotype distributions in RAS and demonstrated that the frequency of the II homozygotes was lower in comparison with other genotypes in women, but not in men with RAS. Some studies have reported that gonadal hormones might affect ACE activity through the *ACE* gene more in women than in men [36] and confirmed evidence that gene regulation of the RA system is strongly influenced by testosterone and estrogen; this may to some extent explain the sexual dimorphism found [37]. The hypothesis that sex steroids alter the activity of the RA system was also confirmed by Sandberg and Ji in their review [38], but much still remains unknown about the molecular mechanisms by which estrogen and androgen alter the system. Therefore, the significant effect of sex found in this study could be a marker for some unmeasured variables that may explain the observed interactions.

Limitations of our study are related to the case–control approach which is vulnerable to population stratification. However, all our subjects were selected from a relatively homogeneous population. The next complicating factor is that the small number of subjects enrolled, especially in the group of RAS patients, may limit the statistical power of this study. Especially, our results of *ACE* gene-by-sex interaction in relation to increased/decreased risk of RAS should be taken carefully. Further, we did not directly study the association between gene polymorphisms and the plasma ACE levels. Nevertheless, the *ACE* genotypes have been previously clearly associated with the plasma and tissue levels of ACE.

In contrast to limitations, the strength of the study is the fact that we focused on five polymorphisms in the *ACE* gene in RAS and that this study provides the first haplotype analysis in this disease. Compared with an isolated study of only one polymorphism, the involvement of the haplotypes may better reveal biological effects caused by an interaction of several polymorphisms in a complex multifactorial disease.

## Conclusion

In summary, this study represents the first evidence of association between *ACE* polymorphisms and RAS in European Caucasians. Although the causal effect of *ACE* variants on the development of RAS is not clear, our results confirm the previous findings of association between *ACE* I/D polymorphism and RAS in a Turkish population. However, further studies in larger independent cohorts are required to prove our results.

## Data Availability

The datasets generated and/or analysed during the current study are available in the DRYAD repository, https://datadryad.org/stash/share/MswOBif8YEiy3HwidH2e4X9w1Pp-UyCi72aygFNiJbg, DOI (https://doi.org/10.5061/dryad.3n5tb2rjw).

## Abbreviations

ACE: Angiotensin I converting enzyme
Ang I/II: Angiotensin I/II
ANCA: Anti-neutrophil cytoplasmic antibody
ASCA: Anti-Saccharomyces cerevisiae antibody
BD: Behçet's disease
CI: Confidence interval
DNA: Deoxyribonucleotide acid
HWE: Hardy–Weinberg equilibrium
I/D: Insertion/deletion
Ig: Immunoglobulin
LD: Linkage disequilibrium
MAF: Minor allele frequencies
N: Number of individuals
NA: Non applicable
OR: Odds ratio
PCR: Polymerase chain reaction
RA: Renin–angiotensin
RAS: Recurrent aphthous stomatitis
SD: Standard deviation
